# Geometry Dynamics of ***α***-Helices in Different Class I Major Histocompatibility Complexes

**DOI:** 10.1155/2015/173593

**Published:** 2015-11-05

**Authors:** Reiner Ribarics, Michael Kenn, Rudolf Karch, Nevena Ilieva, Wolfgang Schreiner

**Affiliations:** ^1^Section of Biosimulation and Bioinformatics, Center for Medical Statistics, Informatics and Intelligent Systems (CeMSIIS), Medical University of Vienna, Spitalgasse 23, 1090 Vienna, Austria; ^2^Institute of Information and Communication Technologies (IICT), Bulgarian Academy of Sciences, Acad. G. Bonchev Street, Block 25A, 1113 Sofia, Bulgaria

## Abstract

MHC *α*-helices form the antigen-binding cleft and are of particular interest for immunological reactions. To monitor these helices in molecular dynamics simulations, we applied a parsimonious fragment-fitting method to trace the axes of the *α*-helices. Each resulting axis was fitted by polynomials in a least-squares sense and the curvature integral was computed. To find the appropriate polynomial degree, the method was tested on two artificially modelled helices, one performing a bending movement and another a hinge movement. We found that second-order polynomials retrieve predefined parameters of helical motion with minimal relative error. From MD simulations we selected those parts of *α*-helices that were stable and also close to the TCR/MHC interface. We monitored the curvature integral, generated a ruled surface between the two MHC *α*-helices, and computed interhelical area and surface torsion, as they changed over time. We found that MHC *α*-helices undergo rapid but small changes in conformation. The curvature integral of helices proved to be a sensitive measure, which was closely related to changes in shape over time as confirmed by RMSD analysis. We speculate that small changes in the conformation of individual MHC *α*-helices are part of the intrinsic dynamics induced by engagement with the TCR.

## 1. Introduction

T cells play a major role in both innate immunity and adaptive immunity. Their surface-bound T cell receptors (TCRs) recognise antigens and thereby detect hazardous organisms or changes inside cells. TCRs recognize short peptide fragments (p) that are bound to major histocompatibility complexes (MHC). Different interactions of TCR/peptide-MHC (TCR/pMHC) are believed to be the basis for distinctive stimuli that lead to and trigger different fates of the T cell, for example, T cell development, thymic selection, lineage commitment, differentiation into effector cells, or memory T cell responses to foreign antigens [1].

MHCs are surface-bound proteins and their role is to present peptide fragments to TCRs, be they self- or alloantigens. To achieve this, MHCs have a cleft that is able to bind peptide fragments. This cleft comprises two *α*-helices and five subjacent lateral *β*-strands forming a sheet or floor of the cleft. TCRs engage peptide MHCs in a diagonal arrangement that seems to be a common mode of interaction across TCRs [2, 3]. The two MHC *α*-helices interact with the TCR complementarity determining regions (CDR) 1 and 2, while the MHC-bound peptide interacts with CDR3, although CDR1 has also been shown to interact with the terminal parts of the peptide. Most of the sequence variability of TCRs is found within CDRs; these regions are also referred to as hypervariable regions. The two MHC *α*-helices are of particular interest as they represent stable secondary structural domains interacting with TCRs.

Adhesion and signaling proteins together with a set of TCR/pMHC complexes form a junction between T cell and an antigen-presenting cell that is referred to as an immunological synapse. It serves as a platform for assembly and segregation of signaling complexes, which cooperatively decide the outcome of T cell activation and effector function. New findings show that this supramolecular complex forms too late to be relevant for initial TCR signaling that happens within seconds after pMHC engagement, with the TCR initiating a tyrosine phosphorylation cascade [4]. Such an early signal may be sufficient to trigger effector function like killer T cell cytolysis of target cells. Stimulation of proliferation, however, requires engagement and signaling for many minutes or even hours [5–8]. It is important to note that *αβ*-TCR itself has no signaling motif but associates with homo- and heterodimeric cluster of differentiation 3 (CD3) subunits, *ζζ*, *εδ*, and *ε* in a noncovalent way. These subunits contain immunoreceptor tyrosine-based activation motifs (ITAMs) that can be phosphorylated and initiate downstream signaling of TCR activation.

Protein structures as found in protein databases (e.g., Protein Data Bank (PDB)) show a static image. In contrast to that, in molecular dynamics (MD) simulations, the protein explores many conformations and allows one to capture its dynamics. Computational immunoinformatics is a well-established, rapidly evolving field [9]. In previous papers [10, 11] we presented a first evaluation of three TCR/pMHC systems that differ only slightly in the MHC amino acid sequence. Macdonald et al. [12] determined binding characteristics and immunogenicity of MHC alleles HLA-B^*∗*^44:02, HLA-B^*∗*^44:03, and HLA-B^*∗*^44:05 in complex with the ABCD3 self-peptide (EEYLQAFTY) and LC13 TCR. HLA-B^*∗*^44:02 and B^*∗*^44:05 trigger an immune response when bound to the LC13 TCR, while HLA-B^*∗*^44:02 does not despite extensive amino acid sequence homology. This renders these HLA alleles an interesting set to study TCR allorecognition. Macdonald et al. [12] determined the X-ray structure of the ternary complex HLA-B^*∗*^44:05, ABCD3 nonapeptide, and LC13 TCR that is accessible on the protein database (PDB, http://www.pdb.org/, PDB-ID: 3KPS). Due to extensive sequence identity we were able to use in silico site-directed mutagenesis to obtain 3D structures of the missing TCR/pMHC complexes. MD simulations in the nanosecond range could possibly show short-lived changes in dynamic behaviour, conformation, propagation of forces, or early activation signals [13, 14].

The aim of the present paper is to put forward adequate tools for identifying and monitoring of conformational changes with possible functional relevance in MHC *α*-helices, and in particular to monitor geometric characteristics of the MHC peptide-binding groove. Here, we present (i) a robust and parsimonious method to find an approximation of a protein's *α*-helical axis, (ii) an evaluation of polynomial degree adequacy in describing bending or hinge movements of particular *α*-helical axes, and (iii) application of polynomial fitting of *α*-helical axis to monitor *α*-helical conformations along MD simulations in different TCR/pMHC immunological complexes.

Our previous studies established the use of polynomials to model *α*-helices in MHC molecules and monitor their dynamic behaviour [15]. To mathematically describe the structural dynamics of MHC *α*-helices at the TCR/pMHC binding interface we first identified those helical regions which were stable and therein those which are close to the protein-protein interface. Then we extracted the *α*-helical axis and finally determined a polynomial that approximates this axis in a least-squares sense.

Various methods to extract a helix axis have been developed [16], including rotational fitting, using C_*α*_ atoms as control points of B-splines [17] or fitting to a helix. We used a fragment-fitting method, based on previous work [16], to locate the axis of *α*-helices as follows: an ideal, linear *α*-helix fragment comprising four C_*α*_ atoms is superimposed on successive pieces of MHC *α*-helices in a least-squares sense. Along the axis of the fitted helical fragment, we adopt points as estimates of the MHC *α*-helix axis and fit a polynomial through these points. From the polynomial, geometric parameters can be derived to monitor conformational changes. Polynomials fitted to the *α*-helical axis can, in principle, be of any degree. However, polynomials of higher order tend to oscillate, adding noise to geometrical quantities computed thereof. We therefore evaluated polynomials of different degrees for their ability to reproduce bending and hinge motions of an *α*-helix with minimum relative error. Between two adjacent *α*-helices, as found in MHC proteins, the polynomials serve to span a ruled surface. This interhelical surface lends itself to derive several quantitative characteristics of shape: (a) total area [18, 19], (b) a profile of interhelical distances along the binding cleft, and (c) heuristic “centre line of the cleft” which may be constructed, along which the surface torsion, that is, a twist or screw of the interhelical surface, can be computed. The latter characterizes the positions and bending of helices relative to each other and defines the geometrical shape of the peptide-binding cleft that is ligated to the TCR. Dynamics in the shape of the protein-protein interface might modulate the TCR/pMHC binding affinity.

## 2. Methods

### 2.1. Homology Modelling of TCR/pMHC Complexes

Three TCR/pMHC systems listed in Table 1 were simulated. The X-ray structure of TCR/pMHC B4405 (number 3 in Table 1) was taken from the PDB (PDB-ID: 3KPS). Structures B4402 and B4403 were engineered by means of in silico site-directed mutagenesis [20] using B4405 as a structural template. We introduced

(i) mutation Y116D to the MHC molecule to get LC13/ABCD3/HLA-B^*∗*^44:02 complex (B4402),
(ii) mutations Y116D and D156L to the MHC molecule to get LC13/ABCD3/HLA-B^*∗*^44:03 complex (B4403).

**Figure 1:**
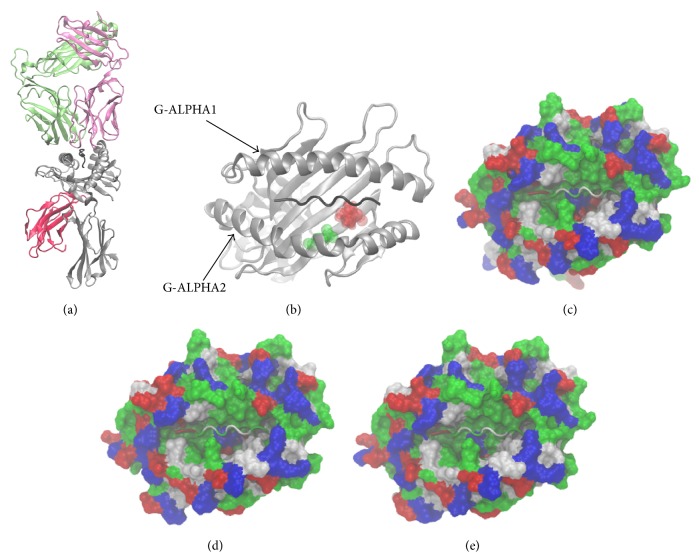
TCR/pMHC. The three HLA molecules studied in this paper are closely related and differ only at amino acid position 116 and/or 156. The X-ray structure of HLA-B^*∗*^44:05 in complex with LC13 TCR and ABCD3 peptide (b) is available from http://www.pdb.org/ (PDB-ID: 3KPS) and was used as a template to model similar systems containing HLA-B^*∗*^44:03 and HLA-B^*∗*^44:02. HLA-B^*∗*^44:05 $\overset{Y116D}{\to }HLA\text{-}B^{*}$44:02 $\overset{D156L}{\to }HLA\text{-}B^{*}$44:03. (a) Cartoon representation of TCR/pMHC system. The TCR comprises two chains (lime and pink). Each chain is made up of a constant domain and a variable domain. The constant domain faces the membrane. The CDR loops 1–3 are highly polymorphic regions that interact with the MHC. Beta-sheets are the main secondary structural element of the TCR. MHC (grey) class I comprises alpha-helices and beta-sheets. Alpha-helices G-ALPHA1 and G-ALPHA2 together with the underlying beta-sheets comprise the peptide-binding pocket and present digested peptide fragments on the cell surface. (b) Cartoon representation of MHC class I. HLA molecule (grey), peptide (black), tyrosine at position 116 (red), and aspartic acid at position 156 (green). (c, d, and e) Surface representation of MHC binding grooves of B4402 (c), B4403 (d), and B4405 (e). Nonpolar residues (white), basic residues (blue), acidic residues (red), and polar residues (green). The ABCD3 peptide is embraced in the peptide-binding groove displayed in cartoon representation. Helix G-ALPHA2 is dominated by alternating acidic and basic residues. The Y116D mutation introduces a negatively charged residue (compare panel (c) with (b): a red spot appears at the right-hand side of the peptide). The D156L mutation substitutes a charged residue with an apolar residue. Structures are taken from the first frame of MD simulations.

See Figure 1(b) for a 3D representation of amino acid positions Y116 and D156. 3D structures were edited and mutations introduced with the Swiss PDB Viewer. The program automatically browses a rotamer library and selects an amino acid rotamer minimizing a scoring function in order to fit the new amino acid in its surrounding and avoid steric clashes with other atoms. All MHC molecules simulated have an amino acid sequence identity of more than 99% and stay in very similar 3D fold. MHC molecules appeared stable during all our MD simulations as seen in RMSD plots in Figure 15.

The full amino acid sequence of HLA-B alleles is accessible from the HLA library (https://www.ebi.ac.uk/ipd/imgt/hla/) and a description of its topology is accessible from UniProt (http://www.uniprot.org/uniprot/Q95365). Not surprisingly, a sequence comparison showed that a transmembrane helix (24 amino acids) and cytoplasmic tail (30 amino acids) are missing from the MHC X-ray structure as plasma membrane structures and flexible protein parts are hard to determine using X-ray crystallography. The LC13 TCR is also missing its transmembrane helix.

### 2.2. Molecular Dynamics Simulations

GROMACS 4.0.7 was used for molecular dynamics simulation. First, water molecules were added to the protein structure, immersing it in an artificial water bath of rectangular form and allowing a minimum distance of 2 nm between the protein and the box boundaries. Second, water molecules were replaced by sodium and chloride ions to yield a salt concentration of 0.15 mol/L and neutralize the protein net charge. Third, the energy of the solvated system was minimized using a steepest descent method and then the system was gradually heated up to 310 K during 100 ps position restraint MD simulation. Finally, MD production runs were done with the LINCS constraint algorithm acting on all bonds using the Gromos 53A6 force field [21]. Hydrogen and fast improper dihedral motions were removed, allowing for an integration step of 5 fs. Van der Waals and Coulomb interactions were computed using a cut-off of 1.4 nm. Long-range Coulomb interactions were computed by Ewald summation. Velocity rescale temperature coupling was set to 310 K and Berendsen isotropic pressure coupling was set to 1 bar. The selection of the force field and MD parameters for pMHCs were evaluated by Omasits et al. [22] and set accordingly.

### 2.3. Finding Dynamically Stable *α*-Helices at the Protein-Protein Interface

As outlined in the introduction, the MHC protein comprises *α*-helices and beta-sheets. We are interested in monitoring C_*α*_ atoms that form stable *α*-helices and are in close contact with the TCR. From MD simulation data we calculated the following:

(i) The relative presence of *α*-helical structure in the protein complexes over the simulation time: we used the DSSP algorithm [23] as implemented in GROMACS to identify secondary structural elements of the protein complex over simulation time. *α*-helices were considered stable if the ratio
  (1) $$\begin{array}{c} \frac{t_{\alpha \text{-}helix}}{t_{sim}}\geq 0.5 \end{array}$$
  with *t*_*α*-helix_ being the time the main chain (backbone atoms plus carbonyl oxygen atoms) meets the DSSP criterion for an *α*-helix and *t*_sim_ being the total simulation time. The cut-off value of 0.5 is justified by the distinctly bimodal distribution (see Figure 11(a)).
(ii) The relative presence of close contacts at the protein-protein interface: C_*α*_ atoms that are less than 1.4 nm apart (i.e., the cut-off for electrostatic interactions in our MD simulations) are defined as being in close contact. Atom-atom contacts between TCR and MHC are defined stable if the ratio
  (2) $$\begin{array}{c} \frac{t_{contact}}{t_{sim}}\geq 0.5 \end{array}$$
  *t*_contact_ is the time during which two atoms are in close contact, and *t*_sim_ is the simulation time. Again, the cut-off value of 0.5 is justified by the distinctly bimodal distribution (see Figure 11(b)). To get the residue-wise relative contact time, we averaged the atom-wise relative contact time (defined in (2)) over all atoms per residue.

**Figure 2:**
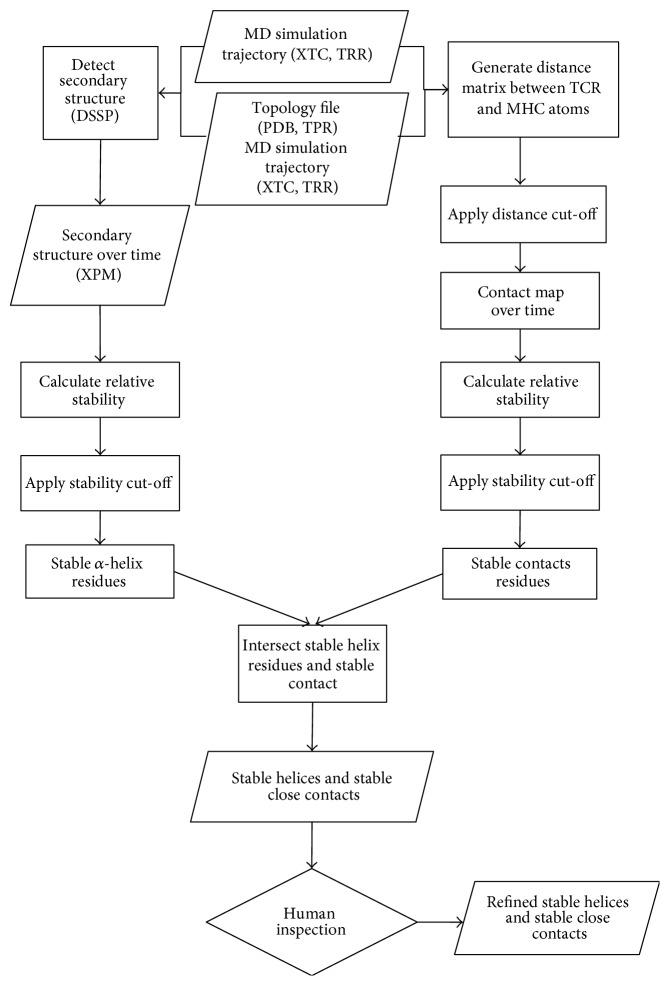
Workflow for selecting stable *α*-helical contact residues. Starting from MD simulation data we calculated the relative presence of *α*-helical structures using the DSSP algorithm (left path) and the relative presence of close contacts over the simulation time (right path). The resulting sets of amino acids are intersected as to yield one list of amino acids that fulfil both criteria: (i) being located within a certain distance to the TCR for more than half of the simulation time and (ii) being part of an *α*-helix for more than half of the simulation time. The process results in a list of amino acids that are stable *α*-helices and stable close contacts. The authors inspected the list in order to rule out the fact that only parts of *α*-helices were selected. Subjecting only parts of a helix to the fragment-fitting method would result in the calculation of a meaningless helical axis.

**Figure 3:**
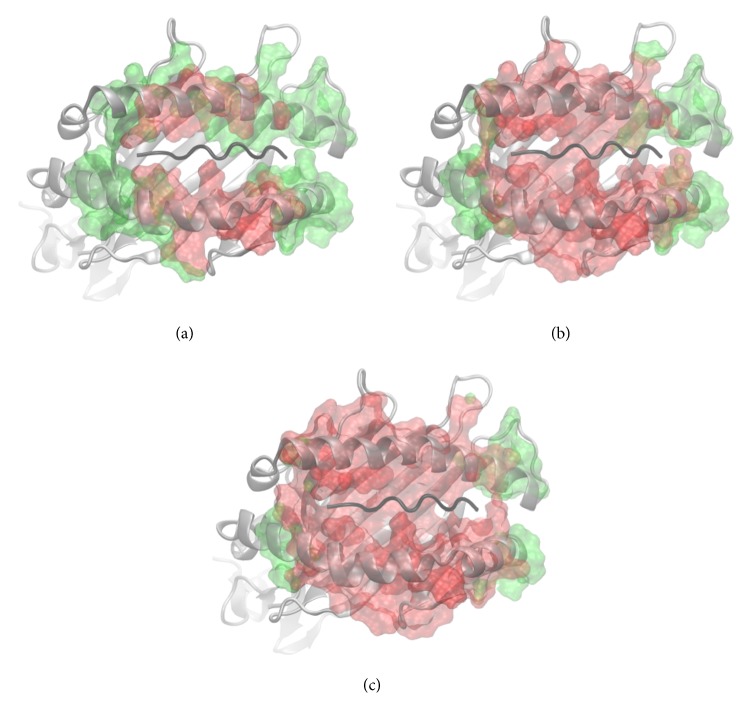
Helical residues and close TCR contacts. The atomic surface in green represents stable *α*-helical amino acids of the MHC that were identified by the method described in Section 2.3. The atomic surface in red represents amino acids in close proximity of the TCR with varying distance cut-offs: (a) 0.8 nm, (b) 1.2 nm, and (c) 1.4 nm. Some parts of the MHC *α*-helices G-ALPHA1 and G-ALPHA2 comprise the protein-protein interface between TCR and pMHC and, as expected, atomic surfaces in green and red overlap. However, not all parts of the *α*-helices belong to the close contacts even when the cut-off is set at 1.4 nm. For calculation of the helical axis we skipped the parts that are not overlapping and not directly interacting with the TCR. Visualization was done with VMD [43]. The contact map was calculated between C_*α*_ atoms to determine which atoms are in close contacts.

The resulting sets of amino acid residues defined by procedures (i) and (ii) were intersected (workflow, see Figure 2; results, see Figure 3) before applying further methods described in Sections 2.4 and 2.5. Dynamically stable helices (green atomic surface in Figure 3) as defined by procedure (i) overlap well with the atoms in close contact of the TCR (red atomic surface in Figure 3) especially with the cut-off set to 1.4 nm. Note that at the end of the MHC's peptide-binding pocket both G-ALPHA1 and G-ALPHA2 exhibit a kink followed by a short *α*-helix. These short *α*-helices are present in the crystal structure, but we found them being unstable during MD simulations as they fold and unfold. We do not consider these helices in our analysis, as they are not in close contact with the TCR.

### 2.4. Approximating the Axis of an *α*-Helix

In order to mathematically describe and quantify *α*-helical geometry and movements, polynomials **c**(*u*) of degree *m* were fitted to the *α*-helices, where **c** represents a vector of 3D coordinates and *u* is the curve parameter. Prior to fitting we extracted C_*α*_ atom coordinates of those amino acids, which fulfil the criterion of stable *α*-helices and close contacts as described in Section 2.3.

According to the structural definition of *α*-helices [24] a model of one ideal *α*-helical turn, that is, a fragment consisting of four C_*α*_ atoms, is constructed, with its axis coinciding with *x*-axis. The coordinates of its *k*th C_*α*_ were assigned as

(3) $$\begin{array}{c} \left(\begin{array}{c} x\\ y\\ z \end{array}\right)=\left(\begin{array}{c} p\cdot \left(k-1\right)\\ r\cdot \cos\left(\varphi \cdot \left(k-1\right)\right)\\ r\cdot \sin\left(\varphi \cdot \left(k-1\right)\right) \end{array}\right)\;k=1,\ldots ,4 \end{array}$$

with pitch *p* = 0.15 nm (advance from one C_*α*_ to the next), helix radius *r* = 0.23 nm, and *φ* = 100 · *π*/180. Along the axis of this helical fragment we consider three axis points, the* initial *(0,0, 0), the* intermediate *(1.5 · *p*, 0,0), and the* final *(3.0 · *p*, 0,0).

**Figure 4:**
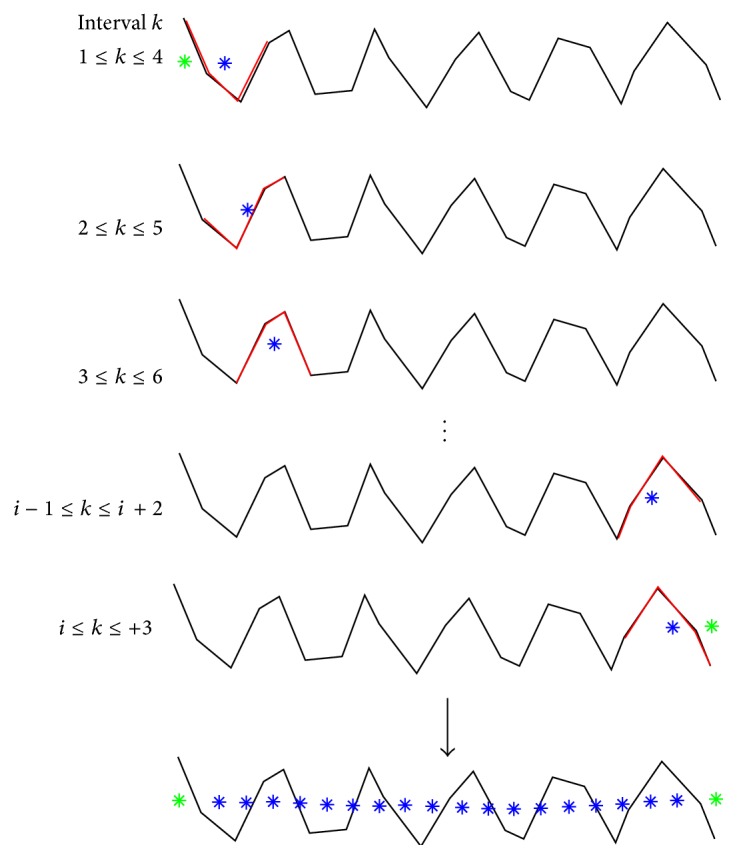
Visualization of the fragment-fitting process. An ideal helical fragment of four C_*α*_ atoms (red) is superimposed on successive pieces of an *α*-helix (grey) in a least-squares sense. Along the axis of the fitted helical fragment we adopt points (blue) as estimates of points on the axis of the MHC *α*-helix. From the very first and last superimposition we adopt one extra point each (green). The sequence of blue and green points (shown at the bottom of the figure) represents an estimate of the axis of the *α*-helix. Subsequently, a polynomial is fitted to these points in a least-squares sense, yielding a simple model of the *α*-helix from which geometric quantities can be derived.

Out of an *α*-helix with *N*  C_*α*_ atoms we pick moving groups of four successive C_*α*_ atoms each, to which we fit the fragment model defined above in a least-squares sense [25]. Along *x*-axis of the fitted fragment model we adopt points as estimates of the axis of the *α*-helix, see Figure 4. From the very first fitted fragment (C_*α*_ atoms 1,…, 4) we adopt two points as points of the helix: the transformed initial axis point as **a**_1_ and the transformed* intermediate* point as **a**_2_. From fragments fitted subsequently we adopt only the respective intermediate points, and from the last fragment (fitted to C_*α*_ atoms *N* − 3,…, *N*), we again adopt 2 points, its “intermediate” as **a**_*N*−2_ and its final point as **a**_*N*−1_. Thus, *N* − 1 points (**a**_1_, **a**_2_,…, **a**_*N*−1_) represent the axis of the *α*-helix.

### 2.5. Fitting the Axis of an *α*-Helix

The points (**a**_1_, **a**_2_,…, **a**_*N*−1_) representing the helical axis are fitted by polynomials **c**(*u*) of degree *m* in a least-squares sense. Separate regression functions *f*_*x*_, *f*_*y*_, and *f*_*z*_ are computed for *x-*, *y-,* and *z*-coordinates:

(4) $$\begin{array}{c} c\left(\;u\;\right)=\left(\begin{array}{c} f_{x}\left(u\right)\\ f_{y}\left(u\right)\\ f_{z}\left(u\right) \end{array}\right); \end{array}$$

for example, for *x*-coordinate,

(5) cxufxu=px,mum+px,m−1um−1+⋯+px,1u+p0.

The total number of parameters for this model is *N*_parameter_ = 3 · (*m* + 1). When fitting the curve, parameter *u* is evaluated at discrete values *u* = 1,2.5,3.5,…, *N* − 1.5, *N* of pitch, corresponding to the positions of estimated points along the helical axis. After the regression has been performed, **c**(*u*) in (5) may be evaluated for arbitrary, continuous values 1 ≤ *u* ≤ *N*, yielding a continuous representation of the helical axis.

Both G-ALPHA1 and G-ALPHA2 helices were modelled in the same way, yielding models **c**_1_(*u*) and **c**_2_(*u*) with equal polynomial degrees *k*. It is well known that (with equidistant data points) interpolating polynomials of too high a degree may exhibit severe oscillations near the ends of the interpolation interval [26]. (Interpolating polynomials actually pass through all data points.) This is also true for approximating polynomials [27]. (Approximating polynomials need not pass through data points but rather approximate the shape of their functional dependence in a least-square sense.) We therefore kept the polynomial degrees as low as possible.

### 2.6. Geometric Quantities

#### 2.6.1. Interhelical Distance and Area of Interhelical Surface

**Figure 5:**
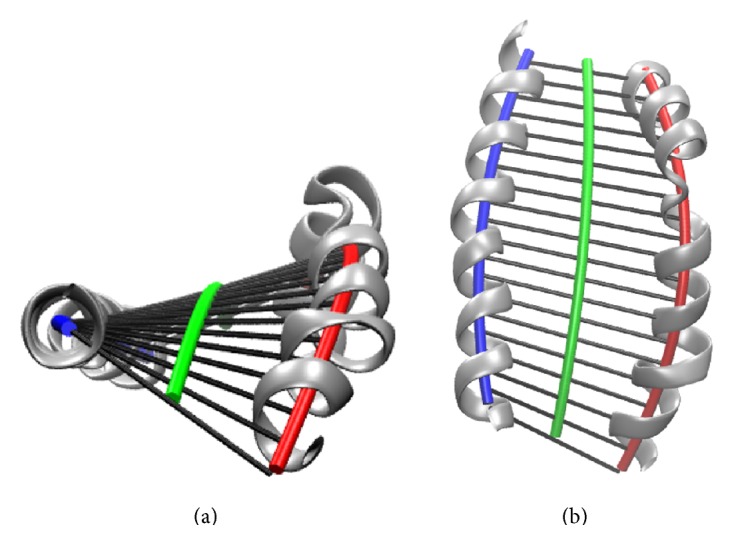
Surface torsion of the interhelical surface. Front view (left) and top-down view (right) on MHC *α*-helices G-ALPHA1 and G-ALPHA2 whose axes are modelled by second-degree polynomials (blue and red). Lines (in grey, called “rulings”) between the polynomials span a ruled surface. Taking the mean coordinates of the lines coloured in blue and red results in the centre line (green). When moving over the surface along the centre line we see that rulings change direction, which can be quantified by a parameter called “surface torsion.” Surface torsion describes the extent and orientation of twist of a surface along a given line, which is the centre line in our case. The surface torsion describes important aspects of the relative orientation of the two helix axes towards each other.

For each polynomial, the curve parameter ranges within 1 ≤ *u* ≤ *N*, with possibly different values (*N*_1_, *N*_2_) for each helix. We consider *L* = min (*N*_1_, *N*_2_) equidistant values of *u*, yielding *L* reference points on each helix model. Connecting corresponding reference points by straight lines yields rulings, **X**(*u*) = **c**_2_(*u*) − **c**_1_(*u*), which span a ruled surface (see Figure 5). (The rule for defining “corresponding” points has to be adopted in a reasonable way but finally remains to some degree arbitrary.) From rulings we estimate distances |**X**(*u*)| between polynomials across the cleft. The resulting polygon mesh was triangulated and interhelical area *A* was calculated, as previously outlined [18, 28]. Each of these quantities may be monitored over time, for example, *A*(*t*); see Figure 9. Respective graphs provide well-defined estimates of changes in width of the intrahelical gap (binding cleft) as a function of both helical position and time. Likewise, median, quartiles, and extreme values of interhelical distances for each *u* and of *A* can be obtained.

#### 2.6.2. Torsion of Interhelical Surface

The ruled surface between both polynomial helix models may bend and wind in various ways. Describing all aspects would call for a comprehensive mathematical treatment in terms of differential geometry, from which we refrain. Instead, we restrict ourselves to describe something like the “torsion of the interhelical surface” in a simple, intuitive way; see Figure 5. (In everyday terminology “torsion” is often called “winding.” It may apply to (curved) lines as well as to surfaces in 3D space. We use both terms in parallel with equal meaning.)

To this end we note that the observer may slide across a surface along various paths and may, in general, observe different values of surface torsion along each of the paths. We notice that torsion in strict mathematical terms is a local characteristic of the surface, and even more, it depends upon the path one takes to inspect the surface. For the sake of simplicity we deliberately adopt the centre line between both helix models:

(6) $$\begin{array}{c} m\left(\;u\;\right)=\frac{1}{2}\left[\;c_{1}\left(\;u\;\right)+c_{2}\left(\;u\;\right)\;\right], \end{array}$$

and let **t**(*u*) be the unit tangent vector of **m**(*u*). Of note, the centre line intersects with each of the rulings. While proceeding along the centre line we inspect the directional change of rulings. This provides us with a particular characterization of the deformation of the interhelical surface. To quantify this intuitive description, we introduce a parameter “surface torsion.” It is based on the change in direction between successive rulings [29] and is determined by the derivative **X**′(*u*) = **c**_1_′(*u*) + **c**_2_′(*u*). (In strict mathematical terms (of differential geometry) this quantity is called “parameter of distribution,” which we think is a very nonintuitive label, prone to be mixed up with probability distributions. Hence, we prefer the more intuitive term “surface torsion” to quantify the winding of a surface while proceeding along a given path (in our case the centre line), not that surface torsion is generally different in different directions, even in the very same point of a surface.) What we call* surface torsion* is given by [29]

(7) $$\begin{array}{c} \tau \left(\;u\;\right)=\frac{\left(X\times X^{\prime }\right)\cdot t}{{\left|X\right|}^{2}}. \end{array}$$

The integral value

(8) $$\begin{array}{c} T=\int_{path\;m\left(u\right)}\tau \left(\;u\;\right)du \end{array}$$

quantifies the relative tilt of the surface between start and endpoint of the path. Note that *τ*(*u*) > 0 indicates right-handed surface torsion, *τ*(*u*) < 0 indicates left-handed surface torsion, and *τ*(*u*) = 0 indicates that the surface at this point is developable and rulings are parallel. (“Developable” means that a sheet of paper could be bended to exactly match the surface; the surface could be “flattened.”) In summary, the definition of the intrahelical surface depends on a reasonable selection of rulings, **X**, and a path, **m**, which are both arbitrary to some extent. Despite these shortcomings in a strict mathematical sense, the concept of torsion, as introduced here, mirrors some essential features in describing the interplay between the shape of *α*-helices and their relative orientation in forming the MHC binding cleft.

#### 2.6.3. Curvature of Helices

Derivatives of each polynomial helix model can be obtained analytically for each value of the parameter *u*, yielding the vectors

(9) c′u=dcdu,c′′u=d2cdu2.

Curvature *κ* is a scalar quantity being per definition positive in Euclidian 3D space and is obtained via [29]:

(10) $$\begin{array}{c} \kappa \left(\;u\;\right)=\frac{\left|c^{\prime }\left(u\right)\times c^{\prime \prime }\left(u\right)\right|}{{\left|c^{\prime }\left(u\right)\right|}^{3}}. \end{array}$$

#### 2.6.4. Construction of a Curved Helix Model and Helix Hinge Model

**Figure 6:**
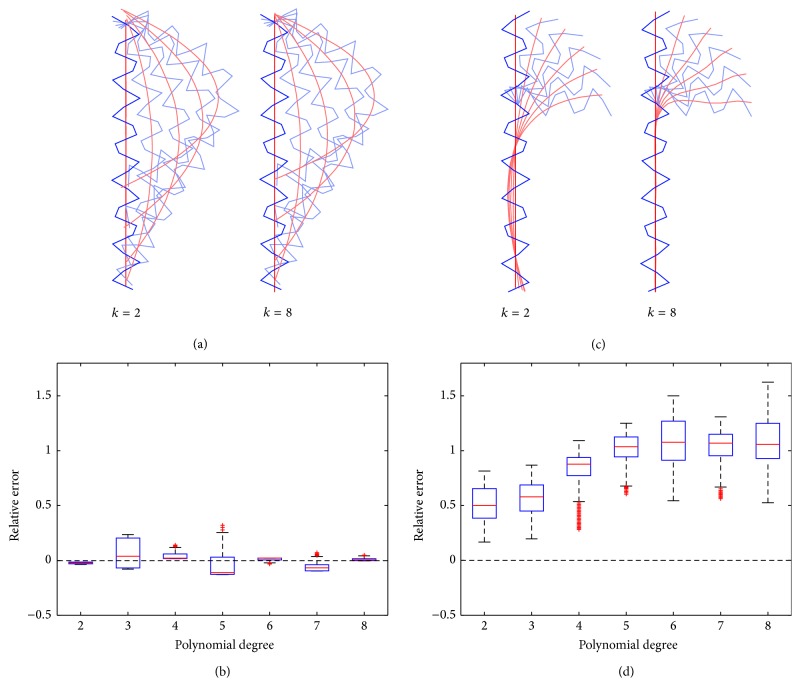
Capturing helix motions with polynomials of different degrees. Panel (a) is an illustration of a helix bending movement. We created a model of an ideal linear helix (blue) comprising only C_*α*_ atoms whose axis is gradually bent by a mathematically well-defined function. From this function we can easily derive the curvature (∫*κ*_bending_^reference^ *du*) and compare it to the curvature we measure (detect) from the polynomial fitted to the helical axis (∫*κ*_bending_^detector^ *du*) that was calculated by the fragment-fitting method. From this comparison we derive the relative error; see (11). Panel (b) shows the relative errors of bending motion for polynomial degrees 1 to 8. Panel (c) is an illustration of a helix hinge movement. We created a model of an ideal linear helix (blue) comprising only C_*α*_ atoms and split it into two parts. One part was 10-atom long and the other part was 20-atom long. Then one part was rotated around a pivotal point as to simulate a hinge movement. The curvature integral of the helical axis was compared to the hinge angle by calculating the relative error. Panel (d) shows the relative errors of hinge motion for polynomials degrees 1 to 8. Polynomials of second degree were found to reproduce the bending and hinge angles with minimal relative errors.

So far we have described how an *α*-helical axis is reconstructed by our newly proposed fragment-fitting method and fitted by a polynomial of a certain degree. From this polynomial, we calculate the curvature integral as described in Section 2.6.3. We wanted to find an optimal degree for these polynomials in order to retrieve conformational deformations with minimal errors. To do so we modelled two different motions such that MHC *α*-helices could perform: a bending motion and a hinge motion (see Figure 6).

To model a bending motion we created a curved helix backbone model with known curvature, as described in detail in Appendix A. Following polynomial representation of the backbone, the curvature integral can be obtained analytically, yielding the reference, ∫*κ*_bending_^reference^ *du* (see (A.2) in the appendix).

To test our method, the curved helix model was subjected to the fragment-fitting method and the resulting helix axis was fitted by a polynomial in a least-squares sense as described in Sections 2.4 and 2.5. The polynomial was evaluated at 100 equidistant points and curvature and the curvature integral were calculated numerically, yielding ∫*κ*_bending_^detector^ *du* (see (10)). As a quality criterion for regaining the correct curvature integral we used the relative error

(11) $$\begin{array}{c} \eta_{bending}=\frac{\int \kappa_{bending}^{detector}du}{\int \kappa_{bending}^{reference}du}-1 \end{array}$$

and evaluated it for polynomial degrees 1 to 8. The results are depicted in Figure 6(b).

To model a hinge motion, we constructed an ideal, linear *α*-helix comprising 31 C_*α*_ atoms. Subsequently, the helix was split into two parts: one C_*α*_ atom was selected and the remaining part of the helix rotated around the selected C_*α*_ atom to model a hinge motion (see Figure 6(c)). The position to split the helix (number of C_*α*_ selected) was varied from C_*α*_ atom 5 to 15. The aim was to examine *α*-helix hinges with two legs unequal in length. From this series of *α*-helical models (different positions of the hinge and varying hinge angles, *α*_hinge_) we then reconstructed the *α*-helical axis using the fragment-fitting method. A polynomial of *k*th order was fitted to the traced *α*-helical axis in a least-squares sense and the curvature integral calculated numerically, yielding ∫*κ*_hinge_^detector^ *du*. The relative error

(12) $$\begin{array}{c} \eta_{hinge}=\frac{\int \kappa_{hinge}^{detector}du}{\alpha_{hinge}}-1 \end{array}$$

was obtained for polynomials of degrees 1 to 8, with 0 ≤ *α*_hinge_ ≤ *π*/2 being the angle between the two parts of the helix.

## 3. Results

### 3.1. Finding the Optimal Polynomial Degree for Monitoring Helix Motions

To monitor deformations of *α*-helices in MD simulations we propose an approximation of the *α*-helical axis using a fragment-fitting method (Section 2.4), fitting the resulting axis by a polynomial in least-squares sense (Section 2.5), and derive geometric quantities from it (Section 2.6). To find an optimal degree of polynomials we applied our method by applying it to modelled *α*-helical bending and hinge motions (Figures 6(a) and 6(b)) and evaluated the polynomial degree that retrieves a certain *α*-helical motion with minimal relative error. For the hinge motion we noticed that relative errors increase with polynomials of higher order. For the bending motion, choosing sixth- or eighth-degree polynomials increases the degree of freedom while at the same time failing to add adequate improvement in relative error. Generally, relative errors in the detection of hinge motions are larger than those for bending motions. The second-order polynomials reproduced the measured quantities of bending and hinge motions with low relative error and hence were adopted for monitoring MHC *α*-helices in all subsequent analyses. We admit that second-order polynomials may fail to model *α*-helical axes in full detail, but it seems appropriate for capturing trends in helical motions and shape during an MD simulation with minimal relative error.

**Figure 7:**
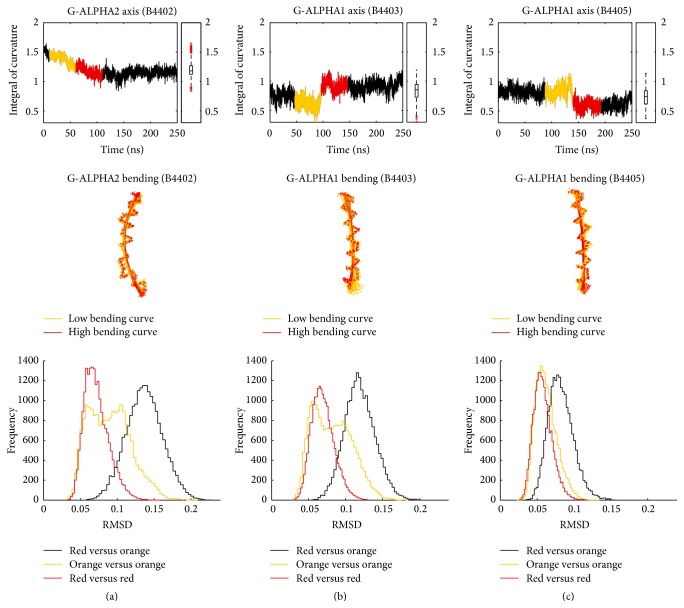
Curvature integral and helix conformations. Three case studies showing the time course of the curvature integral for G-ALPHA1 (b and c) and G-ALPHA2 (a). Upper row shows curvature integral along the 250 ns MD simulation. Phases of increasing or decreasing trends are highlighted in orange and red. Middle row shows 3D conformations of MHC *α*-helices (C_*α*_ atoms) and polynomials derived by fragment-fitting. Colours correspond to the highlighted phases in the upper row. We see that the red bundle of conformations differs from the orange bundle, especially near the ends. Lower row shows that RMSD matrices between helix conformations were calculated and frequencies of RMSD values plotted. Red and orange lines represent frequency distribution of RMSD values between configurations within red and orange phases, respectively. Black lines represent RMSD distributions between configurations in the red and configurations in the orange phases. The difference between lower RMSD within phases and higher RMSD between phases confirms a conformational change between these phases.

We applied the geometric analysis to model MHC *α*-helices of TCR/pMHC complexes B4402, B4403, and B4405. By inspecting the curvature integral for MHC *α*-helices, G-ALPHA1 and G-ALPHA2, three interesting cases could be identified showing changes along the 250 ns MD simulation (Figure 7, panels in upper row). Phases during which the curvature integral changes either abruptly ((b) and (c)) or gradually (a) are highlighted in orange and red. Helix conformations (“bundles”) corresponding to these phases are shown in panels in the middle row. To check if changes in the curvature integral actually indicate a conformational change we calculated RMSD matrices of conformations within and between orange and red helix bundles (panels in lower row). In all three cases, RMSD between phases is distinctly larger (shifted to the right) as compared to RMSD within phases. A shift towards higher RMSD values indicates a conformational change and is seen in all three cases (see Table 2 for median RMSD values). These case studies demonstrate that changes in conformation of *α*-helices during MD simulations can be monitored using second-degree polynomials fitted to helical axes.

### 3.2. Geometric Quantities Characterizing the Shape of MHC *α*-Helices

The MHC peptide-binding groove comprises two *α*-helices that interact with the TCR. Using second-order polynomials, we computed (i) the integral of the curvature of individual MHC *α*-helices, (ii) the area of interhelical surface, and (iii) the surface torsion along the imaginary centre line derived from both polynomials that model MHC *α*-helices for single time steps in MD simulations. Items (ii) and (iii) are geometric properties of the ruled surface. They are used to quantify the geometric relation between the two helices, for example, their relative orientation, and thus capture important aspects of the geometry of the peptide-binding groove, as illustrated in Figure 5.

**Figure 8:**
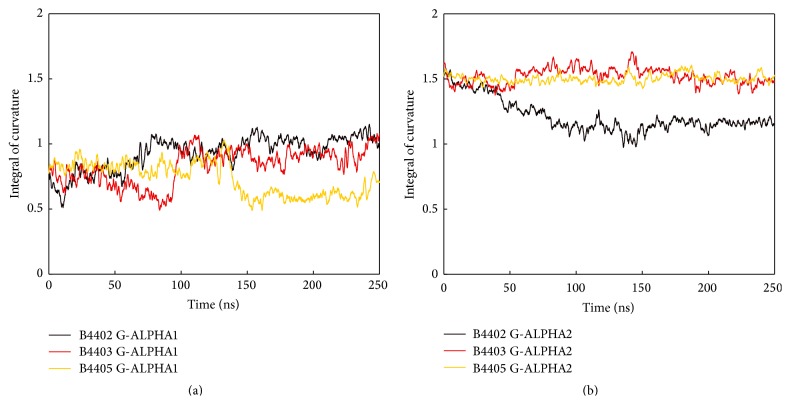
Curvature integral of MHC *α*-helices. Moving average of the curvature integral of MHC helices G-ALPHA1 (a) and G-ALPHA2 (b) along 250 ns MD simulations of TCR/pMHC systems B4402, B4403, and B4405. Curvature of G-ALPHA2 is higher compared to G-ALPHA1 due to its kink. G-ALPHA1 shows greater fluctuations but is comparable between all three TCR/pMHC complexes. G-ALPHA2 of B4402 shows a decreasing trend, consistently reflected in the area of the ruled surface spanned between both MHC *α*-helices (see Figure 9).

Curvature is a local feature of a curve. Considering the curvature integral we obtain a measure of the overall bending of the whole curve [30]. Curvatures of polynomials modelling single helices were integrated and monitored over time as seen in Figure 8. G-ALPHA1 of B4403 and B4405 each undergoes abrupt fluctuations in helical conformation, reflected in the curvature integral, but is stable in the phases in-between. The curvature integral for G-ALPHA1 of B4402 shows a gradually increasing trend. We notice that the curvature integral for G-ALPHA2 is generally higher than that for G-ALPHA1, reflecting the kink near its N-terminal end. The two helical parts of G-ALPHA2 form a hinge. G-ALPHA2 of B4403 and B4405 shows only minor fluctuations and no abrupt changes in the curvature integral indicating that the hinge angle stays stable. For B4402, the hinge angle decreases in the first half of the MD simulation and remains stable thereafter.

**Figure 9:**
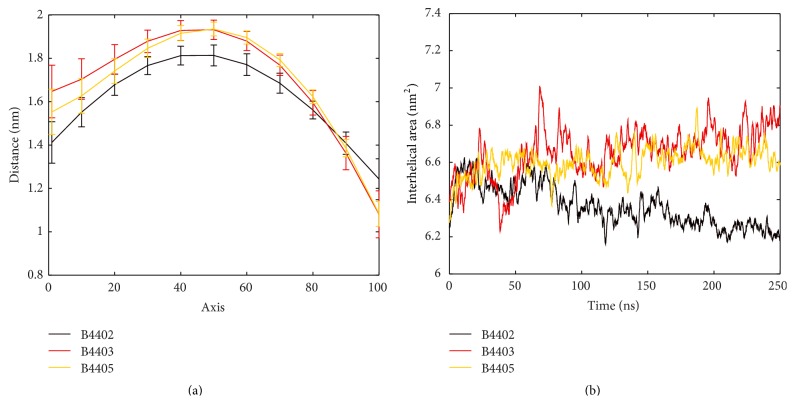
Interhelical distance and area of interhelical surface of MHC *α*-helices. (a) Mean distances between the two MHC *α*-helices as measured at 11 different points along the helices. *x*-axis describes the running parameter of the helices with each helical axis divided into 100 equidistant points. The orientation of the running parameters of both helices is from N-terminus to C-terminus of G-ALPHA1. Distances are measured between corresponding points on each helical axis of G-ALPHA1 and G-ALPHA2. The standard deviation of the mean is shown in the error bars. This distance plot describes the shape and size of the peptide-binding pocket. B4403 and B4405 show a very similar pocket shape. (b) The two MHC *α*-helices span a ruled surface. Moving average of interhelical area along the MD simulation is shown. The magnitudes of interhelical area of B4403 (nonimmunogenic) and B4405 (immunogenic) are similar and slightly increasing, while B4402 shows a declining trend. The curvature integral (Figure 8) for individual helices shows a concomitant bending and relaxing, explaining the shrinkage of interhelical area.

The area of interhelical surface depends on the relative location of both helices. It changes, as the helices drift apart or elongate. It also changes when both helices bend in opposite directions as these amount to a distension of the surface. Whenever helices bend in similar ways in the same direction, interhelical area will be relatively unaffected. Depending on the complexity in shape of the helical axis, second-order polynomials might not adapt to the axis' path accurately. Figure 9 shows an increasing trend of interhelical area for complexes B4403 and B4405, while a declining trend of interhelical area is seen for B4402. These changes occur as the helices of the peptide-binding groove move closer together or further apart, respectively. This is also reflected in the distance of *α*-helical centres of masses (data not shown). However, inspecting Figure 5 clearly demonstrates that the actual shape of an interhelical surface cannot be characterized by a single quantity such as the area of interhelical surface.

A more elaborate descriptor of the shape of a surface is the surface torsion. This parameter describes the change in angle between subsequent tangent planes along a given path, in our case the centre line. High values of surface torsion, regardless of being positive or negative, describe a rapid change in the angle. A pair of helices, each being somehow deformed and both being in varying positions to each other, gives rise to an interhelical surface with a plethora of possible shapes. For describing these shapes geometrically, surface torsion is an important concept, lending itself to quantify the twist in the surface along a prescribed path.

**Figure 10:**
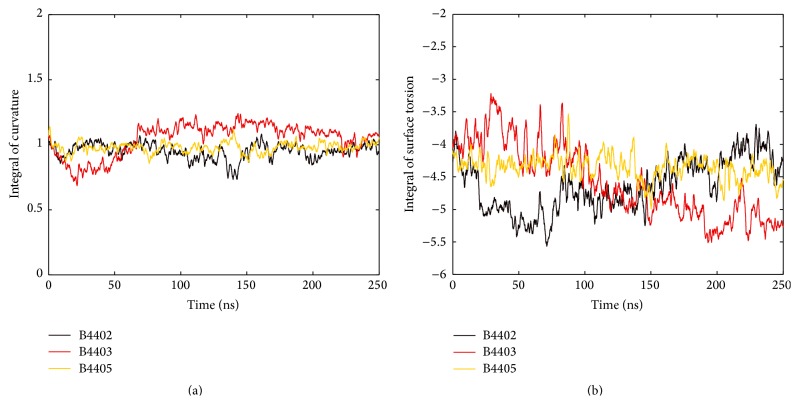
Curvature integral and surface torsion integral of the centre line. (a) The centre line is an imaginary line combining polynomials fitted to axis of G-ALPHA1 and G-ALPHA2 (see Figure 5, green line). The curvature integral of the centre line is stable along the MD simulation for all three TCR/pMHC complexes. (b) The surface torsion integral along the centre line between two polynomials that approximate MHC G-ALPHA1 and G-ALPHA2 is stable for B4405. The other two complexes differ: B4402 shows a drop in the surface torsion integral during the first 60 ns and rises afterwards. B4403 shows a generally decreasing trend.

**Figure 11:**
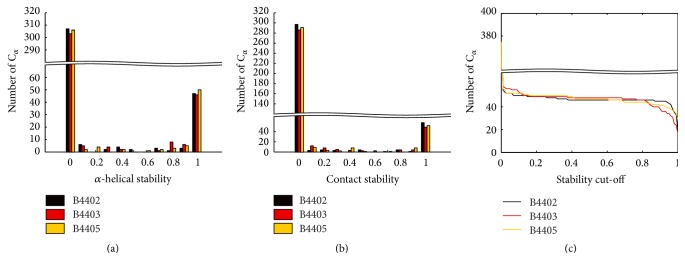
Stability of MHC *α*-helices and protein-protein contacts. (a) Histogram of amino acids that form *α*-helices in the MHC and *β*-2-microglobulin protein complex. *α*-helical stability of 0 means that a given C_*α*_ atom is never part of an *α*-helix during the MD simulation. On the contrary, an *α*-helical stability of 1 means that this C_*α*_ atom is part of an *α*-helix in every time step of the MD simulation and thus is part of a very stable *α*-helix. The histogram shows a distinctly bimodal distribution. (b) Histogram of C_*α*_ atoms forming stable close contacts (atoms being less than 1.4 nm apart) at the protein-protein interface. The distribution is also distinctly bimodal. Contact stability of 0 means that a C_*α*_ atom never forms a close contact during the MD simulation. A contact stability of 1 means that this C_*α*_ atom forms very stable contacts throughout the MD simulation. (c) The number of stable residues on *y*-axis is calculated by intersecting both sets of stable helix C_*α*_ atoms and stable close contacts C_*α*_ atoms. In Section 2.3, we claim that, due to the distinctly bimodal distributions, neither stable *α*-helices nor the number of close contacts is insensitive to the choice of the cut-off. The resulting number of stable residues will roughly stay constant for a wide range of cut-off values (from 0.2 to 0.8), therefore justifying our choice of 0.5 as the stability cut-off.

For B4402, surface torsion along the centre line (see Figure 5(a)) is left-handed most of the time, reaching a minimum (ca. −2, data not shown) at half of its length. Positive values of surface torsion (ca. 0.2, data not shown) are seen only in rare cases and near both surface termini. The ruled surface of the peptide-binding cleft at the N-terminus of G-ALPHA1 appears to be more stable for B4405 and B4403 than for B4402. Similar trends are seen in the surface torsion integral, see Figure 10. It is stable for B4405, but trends are observed for the other two complexes: B4402 shows a drop in the surface torsion integral during the first 60 ns followed by a gradual rise. B4403 is rather stable during the first 60–70 ns and then shows a gradual decrease in surface torsion.

**Figure 14 fig14:**
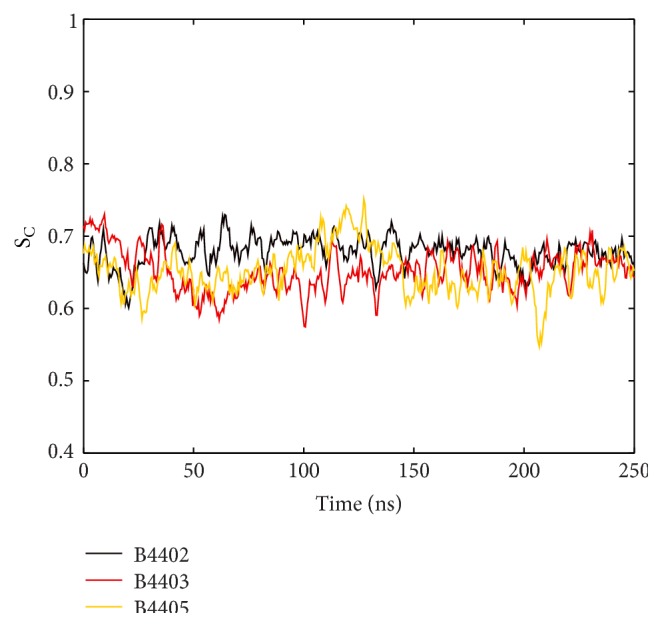
Shape complementarity of TCR/pMHC interface. Lawrence and Colman [31] introduced shape complementarity statistics comparing the surface normal alignment on dots from molecular protein-protein surfaces generated according to Connolly [44]. S_C_ is a measure of how good two protein surfaces fit together. It assumes values between 0 and 1, with 1 indicating a perfect fit. S_C_ is stable and similar for all three TCR/pMHC complexes along 250 ns MD simulations.

**Figure 15 fig15:**
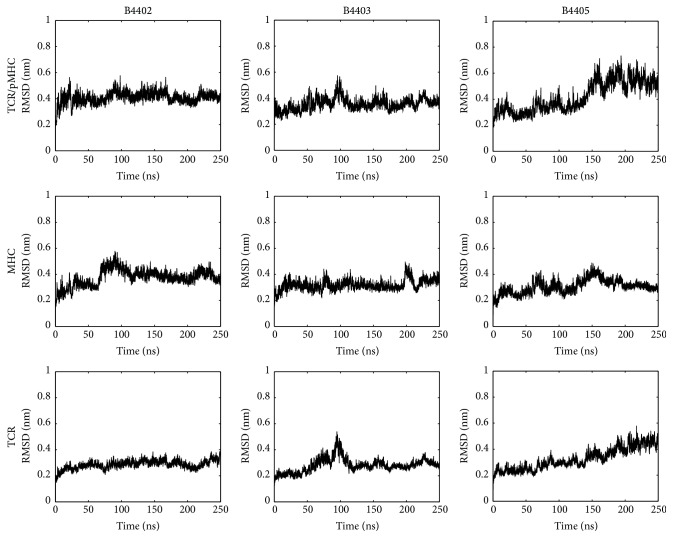
Root mean square deviations. Root mean square deviations of TCR/pMHC systems B4402, B4403, and B4405. Superposition of successive frames was done with respect to protein C_*α*_ backbone of the first frame of the simulation (nonprogressive fitting) and RMSD was calculated between protein C_*α*_ backbones. Row TCR/pMHC shows that the whole protein system, TCR/pMHC, was fitted to itself and RMSD calculated for the whole protein. Row MHC shows that TCR was fitted to itself and RMSD calculated for TCR. Row TCR shows that MHC was fitted to itself and RMSD calculated for MHC. Results for B4405 indicate that 250 ns of simulation time does not suffice to sample the whole phase space, which is a common finding for such large proteins. RMSD time courses for B4402 and B4403 do not explicitly indicate nonstationary behaviour. They indeed show slower and smaller growth of RMSD over time than does B4405, also indicating their stability as a molecule (despite two point mutations introduced). As noted before, the present work intends to delineate techniques for modelling geometries of MHC components and does not aim at statistical comparisons between the motions of different HLA alleles. Ergodicity is hence not a vital issue; see the discussion in Schreiner et al. [45].

We also looked at the shape complementarity (S_C_) (Lawrence and Colman introduced a method to measure how well the surfaces of two proteins at their interface match [31]. The parameter output by this method is called shape complementarity (S_C_).) of the protein-protein interface surface and backbone C_*α*_ RMSD. We found S_C_ to be stable for all three TCR/pMHC systems (see Figure 14). As S_C_ is unaffected by deformations of the binding cleft (as reflected by surface torsion, see above) we conclude that TCRs follow the conformational changes of the MHC surface. RMSDs of B4402 and B4403 reach a low plateau after a few nanoseconds and remain stable thereafter. RMSD of B4405 shows an increasing trend over 250 ns simulation time, rising from approximately 0.3 nm to 0.6 nm; see Figure 15.

## 4. Discussion

The mechanism of TCR activation is still controversially debated and several models have been proposed [32–38]. A recent work of Dustin and Depoil [34] summarizes new insights into the function of the T cell synapse. The authors grouped T cell synapse into three interactive layers including interactions of receptors, a signaling layer, and a cytoskeleton layer, all contributing to TCR activation, regulation, and fine-tuning of signaling and responses. Conformational changes of the TCR complex have been demonstrated to be relevant for signaling of the TCR [39]. Association of CD3 proteins to the TCR/pMHC complex is necessary to transmit the activation signal to intracellular signaling molecules [40]. Evidence suggests that TCR conformational changes are required for full activation, but there are certain signaling pathways that can also be activated in the absence of conformational changes [41]. The three TCR/pMHC complexes analysed in this work differ only by one or two amino acids in the MHC molecule. HLA-B^*∗*^44:05 (B4405) and HLA-B^*∗*^44:02 (B4402) MHC types trigger LC13 TCR activation in the presence of the ABCD3 self-peptide. Surprisingly HLA-B^*∗*^44:03 (B4403) does not trigger TCR activation.

To characterise the dynamics of the MHC antigen binding cleft, we applied (similar to the methods reported by Christopher et al. [16]) a fragment-fitting method to model stable *α*-helical regions that are in close proximity (≤1.4 nm) to the TCR and monitored their geometric parameters. However, it is known that geometric quantities derived from polynomial approximations may vary substantially depending on the polynomial degree chosen [10]. To select an appropriate polynomial degree, we tested the ability of polynomials with different degrees to retrieve predefined parameters of helical motions and deformation. In a simplified model, we tested the ability of the fragment-fitting method to reproduce the curvature integral of helical bending and hinge motions. We found that second-order polynomials are best suited to model these *α*-helical motions with low relative error. The curvature integral derived from polynomials of individual *α*-helices can be related to conformational changes of *α*-helices. Between the two MHC *α*-helices a ruled surface can be spanned, of which we computed the area as an estimate for the size of the peptide-binding cleft. We also calculated the surface torsion along an imaginary centre line characterizing the orientation of both *α*-helices relative to each other. We applied this method to MD simulations of three TCR/pMHC complexes. However, we were not able to find correlations between immunogenicity and certain patterns in the *α*-helical movements.

### 4.1. Limitations

A limitation of the current analysis is that the ruled surface between the MHC *α*-helices that we use to model the MHC surface presented to the TCR does not consider the shape and dynamics of the peptide that lies between the two helices. We cannot assume that phase space has been sampled comprehensibly for these large molecules. Stepwise fluctuations in measured variables are visible (see Figure 7(b), upper row, and Figure 15). Also, even with highly optimized simulation performance of 15 ns/day on 1024 computing cores of the IBM BlueGene, statistics to discriminate between different simulated systems is not feasible. Enhanced sampling techniques and adequate collective variables might be useful to identify adequate collective variables for such systems. Interpretation of single simulations should therefore be done with caution. Signaling may involve a series of other proteins of the immunological synapse and interactions that are not considered in these simplified TCR/pMHC models. Interactions between TCR and pMHC take place between two cells that are in close contact to each other. It has been shown that plasma membrane lipids affect the activity of signaling networks [42] and some models of TCR/pMHC interaction propose involvement of the plasma membrane [33].

### 4.2. Conclusion

In this work, changes of MHC shape and their dynamics were quantified. We applied the quantification method to three large TCR/pMHC complexes, due to their size being accessible by MD simulation studies only since recently. We saw that MHC *α*-helices undergo rapid changes in conformation by either bending motion or hinge motion. Surface torsion used for characterizing the MHC surface presented to the TCR is stable in B4405, which is the most immunogenic complex. We speculate that rapid changes in helical conformation are part of the intrinsic dynamics of MHCs when engaging with TCRs.

Though we were not able to find a clear correlation between immunogenicity and certain patterns in the *α*-helical movements, we could demonstrate that single amino acid polymorphisms in the MHC seem to have a subtle influence on the helixes' shape dynamics and that it would be interesting to apply the same method in the case of peptide polymorphism.

In summary, the present work demonstrates the feasibility and reliability of deriving shape parameters from simulation data. Next, the influence of the detected small conformational changes on the microscopic dynamics will be investigated to clarify their relation to the biological functions of the complexes of interest. Conclusions regarding functional differences between TCR/pMHC systems characterized by a single-residue polymorphism certainly require advanced sampling techniques in order to sample the conformational phase space appropriately for molecules of this size. Future studies might investigate if the small conformational changes in MHC *α*-helices transmit forces to the TCR.

## Figures and Tables

**Figure 12 fig12:**
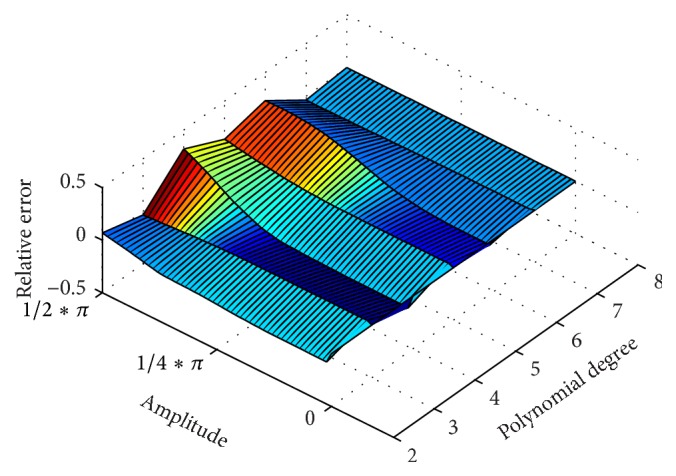
Curvature integral as a function of helical bending. In order to model a bending motion, an ideal linear helix comprising 31 C_*α*_ atoms is distorted by bending its imaginary, linear axis along a cosine function. The curvature of the imaginary axis is known and its integral serves as the reference for the amount of bending. We compare this reference curvature integral with that derived via our fragment-fitting method by calculating the relative error. An ideal method would show a very close to linear correlation between the reference and the measured value. Polynomials of third and fifth degree show the highest relative error, especially for large magnitudes of bending. Sixth-order polynomials or polynomials of higher order look quite promising regarding relative error. Polynomials of higher order were ruled out because of overfitting and the fact that spurious terminal oscillations might occur. Second-order polynomials show a well behaved, close to linear dependence and were therefore adopted to model *α*-helices of MHCs.

**Figure 13 fig13:**
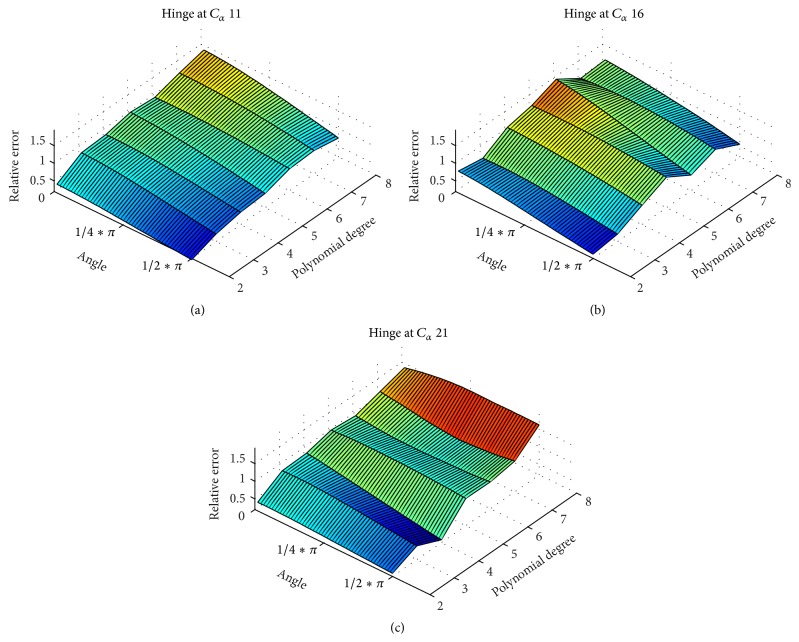
Curvature integral as a function of hinge movement. The relative error in retrieving the correct hinge angle is plotted against helix hinge angle and polynomial degree. To model the hinge motion, a kink of varying angle was introduced to an ideal linear helix comprising only C_*α*_ atoms (31 atoms). Images (a), (b), and (c) show the same data for different positions of the kink in the helix. We refer to the kink angle as the signal we want to measure. We compared the signal to the curvature integral of the polynomial fitted to the helical axis by calculating the relative error. An ideal method would show a linear correlation between signal and the measured value. We see that polynomials of higher order show a higher relative error and overestimate the magnitude of the kink. We also see that the position of the kink modulates the relative error. Second-order polynomials have a nearly linear dependency and were therefore adopted to model *α*-helices of MHCs.

**Figure 16:**
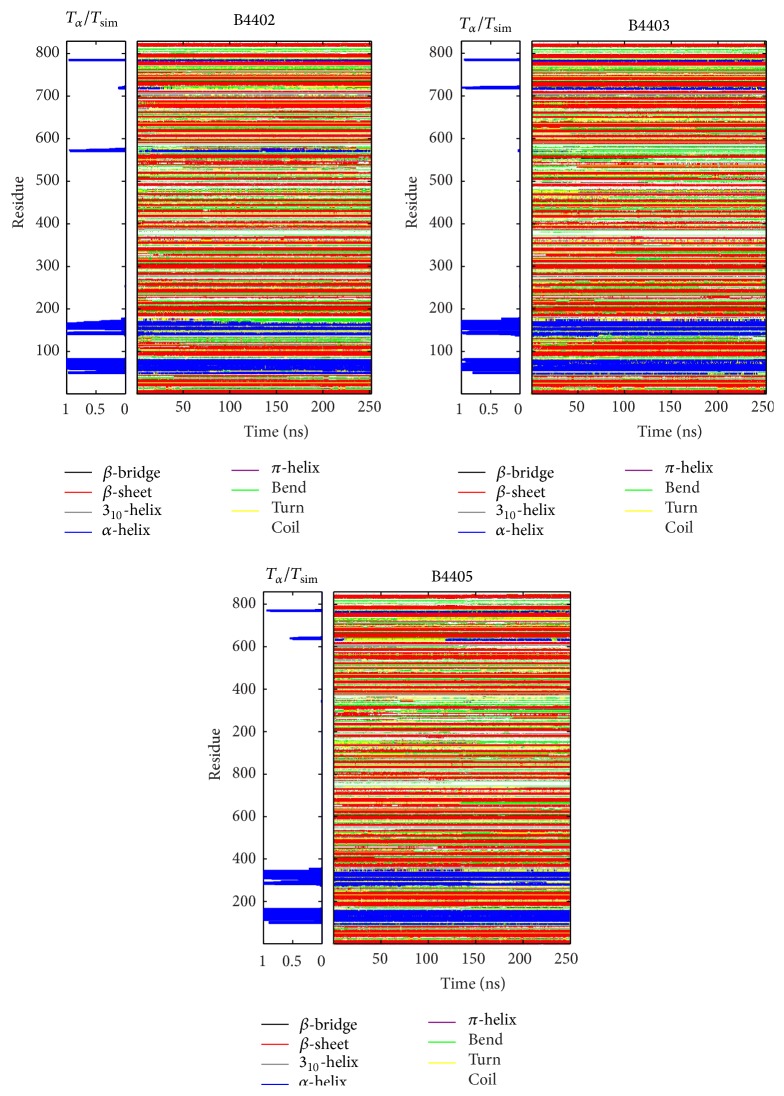
Dynamics of secondary structure. The TCR/pMHC systems B4402, B4403, and B4405 comprise 829 amino acids. The following list shows which residues belong to which protein: MHC, residues 1–276; *β*-2-microglobulin, residues 277–375; ABCD3 peptide, residues 376–384; TCR *α*, residues 384–584; TCR *β*, residues 585–825. The graph on the right-hand side displays the structural behaviour of amino acid residues along the simulation time. Different secondary structural elements are assigned different colours as shown in the legend. Secondary structures are stable along the 250 ns MD simulation for all three TCR/pMHC systems. The graph on the left-hand side displays the relative simulation time that an amino acid residue is part of an *α*-helix. Extended and stable *α*-helices in these TCR/pMHC systems are only present in the MHC molecule.

**Table 1:** Molecular dynamics simulations.

Number	Molecular system (TCR/peptide/MHC)	Simulation length
1	LC13 TCR/ABCD3/HLA-B^*∗*^44:02 (B4402)	250 ns
2	LC13 TCR/ABCD3/HLA-B^*∗*^44:03 (B4403)	250 ns
3	LC13 TCR/ABCD3/HLA-B^*∗*^44:05 (B4405)	250 ns

**Table 2:** Median RMSD values of case studies.

	Orange	Red	Red versus orange
	Median RMSD	Median RMSD	Median RMSD
B4402 G-ALPHA2	0.0870 nm	0.0682 nm	0.1350 nm
B4403 G-ALPHA1	0.0789 nm	0.0671 nm	0.1170 nm
B4405 G-ALPHA1	0.0592 nm	0.0556 nm	0.0794 nm

We compared helix conformations between different phases of the simulation, that is, before and after an inflection point or continous decrease/increase in the curvature integral (see Figure 7, upper row). Median values of the RMSD distribution are shown in this table.

## Appendices

### A. Construction of a Curved Helix Model

The backbone is modelled along a cosine curve. We compute equidistant points along the curve and create C_*α*_ atoms with constant normal distances to the backbone. Angles between successive C_*α*_ atoms were set to *β* = 100°. Formally, coordinates along the axis of the helix are given by

(A.1) x=x=t y=acos⁡t z=0, 

where *a* represents the maximum elongation (amplitude) of the curved helix, compared to *a* = 0, corresponding to a straight model. Increasing *a* in a stepwise fashion generates models of increasing curvature.

For the curvature we obtain

(A.2) κt=asin⁡t1+a2sin2⁡t3/2,κ0=a.

In order to keep the length of backbones constant, the limits for parameter *t* have to be adjusted appropriately; *t* ∈ [−*b*, *b*]. In fact *b* = *b*(*a*) is chosen to make the arc length equal to 1:

(A.3) $$\begin{array}{c} \overset{b}{\underset{-b}{\int }}\sqrt{1+a^{2}\sin^{2}t}\;dt=1. \end{array}$$

By varying *a* within 0 ≤ *a* ≤ *π*/2 different models were created. Curvature decreases with decreasing *a*; in particular *a* = 0 corresponds to a straight line without curvature. In order to find the appropriate values for *b* = *b*(*a*) with *a* > 0, an elliptical integral (see (A.3)) has to be solved. To find *N* equidistant points along the backbone **x**_*n*_ = (*x*_*n*_, *y*_*n*_, *z*_*n*_) = (*x*(*t*_*n*_), *y*(*t*_*n*_), *z*(*t*_*n*_)), for *n* = 1,…, *N*, we proceed, similar to the normalization of the arc length, via numerically solving the elliptical integral:

(A.4) $$\begin{array}{c} \overset{b}{\underset{-b}{\int }}\sqrt{1+a^{2}\sin^{2}t}\;dt=\frac{n-1}{N-1}. \end{array}$$

In the next step, C_*α*_ atoms are positioned in distance *r* to the backbone and successively rotated by *β* = 100°. The tangent vector of unit length at position **x**_*n*_ is given by

(A.5) $$\begin{array}{c} r_{n}=\frac{1}{\sqrt{1+a^{2}\sin^{2}t_{n}}}\left[\begin{array}{c} 1\\ -a\sin t_{n}\\ 0 \end{array}\right]. \end{array}$$

Next, the radius *r* of *α*-helical turns around the axis is set in proper relation 0.23 nm/0.15 nm to the total length of the helix:

(A.6) $$\begin{array}{c} r=\frac{0.23}{0.15}\frac{1}{N-1} \end{array}$$

and the vector **s**_*n*_ to be rotated is given by

(A.7) $$\begin{array}{c} s_{n}=\frac{r}{\sqrt{1+a^{2}\sin^{2}t_{n}}}\left[\begin{array}{c} -a\sin t_{n}\\ -1\\ 0 \end{array}\right]. \end{array}$$

The point **x**_*n*_ + **s**_*n*_ is rotated around the axis **r**_*n*_ by angles *β*_*n*_ = *β*_0_ + (*n* − 1)*β* to create coordinates of successive C_*α*_ atoms (*n* = 1,…, *N*). Finally, all coordinates are scaled via **x** → **x** · *N* · 0.15 nm to arrive at proper dimensions. Thus, we obtain a chain of C_*α*_ atoms rotated in a clockwise manner to represent a curved right-handed helix along a predefined, cosinusodial backbone.

The curved helix model has a few limitations. (i) Due to the curvature of the cosine arc and the construction of C_*α*_ atoms with normal distances, the typical distance between successive C_*α*_ atoms is not maintained. (ii) We refrained from constructing the full backbone also including nitrogen, oxygen, and hydrogen atoms and restricted the model to C_*α*_ carbon atoms only. This seems justifiable, however, since the fragment-fitting algorithm also takes into account C_*α*_ atoms only as does the DSSP algorithm and the calculation of close contacts. Therefore, modelling the full backbone would not add more information to the model. (iii) The cosine arc is planar and hence cannot incorporate any torsion within the curved helix model.

### B. Additional Data

See Figures 11 [Fig fig12] [Fig fig13] [Fig fig14] [Fig fig15]–16.
